# Optoacoustics delineates murine breast cancer models displaying angiogenesis and vascular mimicry

**DOI:** 10.1038/s41416-018-0033-x

**Published:** 2018-03-26

**Authors:** Isabel Quiros-Gonzalez, Michal R Tomaszewski, Sarah J. Aitken, Laura Ansel-Bollepalli, Leigh-Ann McDuffus, Michael Gill, Lina Hacker, Joanna Brunker, Sarah E. Bohndiek

**Affiliations:** 1 Department of Physics, University of Cambridge, JJ Thomson Avenue, Cambridge, CB3 0HE UK; 2 Cancer Research UK Cambridge Institute, University of Cambridge, Robinson Way, Cambridge, CB2 0RE UK; 3 Department of Histopathology, Addenbrooke’s Hospital, Cambridge University Hospitals NHS Foundation Trust, Hills Road, Cambridge, CB2 0QQ UK

**Keywords:** Tumour biomarkers, Cancer microenvironment

## Abstract

**Background:**

Optoacoustic tomography (OT) of breast tumour oxygenation is a promising new technique, currently in clinical trials, which may help to determine disease stage and therapeutic response. However, the ability of OT to distinguish breast tumours displaying different vascular characteristics has yet to be established. The aim of the study is to prove OT as a sensitive technique for differentiating breast tumour models with manifestly different vasculatures.

**Methods:**

Multispectral OT (MSOT) was performed in oestrogen-dependent (MCF-7) and oestrogen-independent (MDA-MB-231) orthotopic breast cancer xenografts. Total haemoglobin (THb) and oxygen saturation (SO_2_^MSOT^) were calculated. Pathological and biochemical evaluation of the tumour vascular phenotype was performed for validation.

**Results:**

MCF-7 tumours show SO_2_^MSOT^ similar to healthy tissue in both rim and core, despite significantly lower THb in the core. MDA-MB-231 tumours show markedly lower SO_2_^MSOT^ with a significant rim–core disparity. Ex vivo analysis revealed that MCF-7 tumours contain fewer blood vessels (CD31+) that are more mature (CD31+/aSMA+) than MDA-MB-231. MCF-7 presented higher levels of stromal VEGF and iNOS, with increased NO serum levels. The vasculogenic process observed in MCF-7 was consistent with angiogenesis, while MDA-MB-231 appeared to rely more on vascular mimicry.

**Conclusions:**

OT is sensitive to differences in the vascular phenotypes of our breast cancer models.

## Introduction

Management of breast cancer has improved significantly in the last two decades.^1^ Nonetheless, the high heterogeneity of the disease means some subtypes have good prognosis while others lack a successful treatment. As a result, breast cancer is still the third most common cause of cancer-related death in the EU.^2^ The different subtypes of breast cancer^3^ reflect different aspects of tumour biology, such as cell of origin, hormone susceptibility and receptor status. Factors such as angiogenesis, inflammatory and immune responses, and oxidative stress show significant cross-talk^4,5^ and play an important role in breast cancer progression and response to therapy.^3,6–8^ In particular, angiogenesis is considered a rate-limiting step in breast cancer progression and holds prognostic significance.^9^

Imaging represents the standard-of-care for breast cancer detection and monitoring. X-ray and ultrasound are normally used together to improve the detection of the lesions, with magnetic resonance imaging (MRI) assisting with delineation of benign and malignant masses.^10^ However, there is presently no validated imaging technique to measure the functional effects of angiogenesis incorporated in the routine medical diagnosis of breast cancer.

In identifying a candidate imaging technique to overcome this limitation, intrinsic sensitivity to the functional effects of angiogenesis, including changes in oxygen supply and demand, hypoxia and perfusion, is preferable. The majority of existing clinical techniques need exogenous contrast agents: contrast-enhanced ultrasound with microbubbles for perfusion and angiogenesis; dynamic contrast-enhanced MRI with gadolinium for perfusion; and H_2_^15^O or FMISO positron emission tomography for perfusion and hypoxia respectively.^11,12^ Potential for associated toxicity and side effects of contrast agents limits the recurrent use of these in the same patient.^13^ Some imaging techniques are not dependent on contrast agents, including Doppler ultrasound, blood oxygen level dependent (BOLD) and oxygen-enhanced (OE) MRI, but they lack sensitivity.^14,15^ Imaging biomarkers of tumour oxygenation^16^ could assist with: selection of appropriate therapies; definition of ‘windows’ for combination therapy; monitoring therapeutic response and reducing healthcare costs of targeted therapy.^17,18^

Optoacoustic tomography (OT) is an emerging imaging modality poised for clinical translation that combines the high contrast of optical imaging with the spatial resolution of ultrasound.^19^ The absorption of pulsed laser energy in tissues generates pressure waves that can be detected by ultrasound transducers. OT is intrinsically sensitive to oxy- and deoxy-haemoglobin, adipose tissue and water content,^20^ enabling detailed characterisation of tumour angiogenesis. Clinical OT operates at up to 5 cm depth in tissue.^21^ Early results characterising breast lesions in clinical trials have shown increased haemoglobin content of lesions compared to normal breast parenchyma.^22,23^ The application OT using haemoglobin and other endogenous chromophores such as melanin or fat suggests that this technique this broad potential not only in the breast, but also for studying other superficial tumour types and beyond cancer, for example in inflammatory diseases.^24–26^ However, despite initial promise, there remain some limitations, one of them importantly being validation of the relationship between OT image features and underlying tumour biology.

To accelerate clinical translation of optoacoustic imaging as a routine diagnostic and monitoring tool, it is imperative to overcome this limitation and better establish the potential of OT, in our case, in clinical breast applications. We have studied the angiogenic microenvironments generated in two breast cancer xenograft models: MCF-7 is an oestrogen-dependent tumour and MDA-MB-231 is an aggressive, oestrogen-independent tumour.^27^ Here we confirm that the distinct angiogenic properties of these tumours can be sensitively detected through OT features and explain our findings through detailed characterisation of the tumour vascular phenotype.

## Material and methods

### Cell lines

The human adenocarcinoma cell lines MCF-7 (Oestrogen Receptor+, OR+) and MDA-MB-231 (Oestrogen Receptor−, OR−) were obtained from the Cancer Research UK (CRUK) Cambridge Institute Biorepository from the University of Cambridge. The experiments were performed when cells were between passage 20–25 for both MCF-7 and MDA-MB-231. Authentication using Genemapper ID v3.2.1 (Genetica) by STR Genotyping (1/2015) showed 100% match with the reference sequence in both cases. Cells were maintained in DMEM supplemented by 10% of FBS at 37 °C in 5% CO_2_. Oxygen consumption was measured using the MitoXpress Xtra Oxygen Consumption assay (Supplementary Information).

### Matrigel culture assay

A 24-well plate was pre-coated with Matrigel (growth factor reduced, SLS) and incubated for 30 min at 37 °C. The MCF-7 or MDA-MB231 cells were added to the pre-coated plate at 10^5^ cells per well in DMEM-F12 medium supplemented with 2% FBS. Images were captured after 24 h using an inverted microscope (Nikon Eclipse TS100).

### In vivo models

All animal procedures were conducted in accordance with project and personal licenses, reviewed by the Animal Welfare and Ethical Review Board at the CRUK Cambridge Institute, and issued under the United Kingdom Animals (Scientific Procedures) Act, 1986. All the procedures meet the standards required by the UKCCCR guidelines.^28^ Seven-week-old immunodeficient female nude (BALB/c nu/nu) mice (*n* = 20; Charles River) were inoculated orthotopically in the mammary fat pad of both flanks with 10^5^ cells (either MCF-7, *n* = 10 or MDA-MB-231, *n* = 10, random group assignment) in a final volume of 100 μL of 1:1 DMEM (GIBCO) and matrigel (BD). Power calculations to determine group size could not be performed in the first instance due to absence of previous data with this particular model and imaging modality, therefore the group size was based on our previous experience conducting in vivo optoacoustic tomography studies in other cell-line derived mouse tumour models.^29^ For MCF-7, endogenous oestrogen levels were supplemented by surgical implantation of oestrogen pellet (0.72 mg/pellet, 90 days release; Innovative Research of America) in the scruff of the neck. In the MDA-MB-231 group, one mouse did not develop any tumours and three mice developed only one tumour; in the MCF-7 group, one mouse developed one tumour only. All tumour-bearing animals were entered into the study. For the tissue analysis, four animals were excluded from the MCF-7 cohort due to side effects caused by the oestrogen pellet (bladder obstruction and skin rash with scabs). Otherwise, all tumours were included in all analyses. Animals were kept in hermetic cages with individual air supply through an EPA filter to guarantee sterile conditions, in 12/12 h ON/OFF light cycles, with enriched environment and food and water ad libitum.

All data acquisition was performed unblinded. Mice were imaged weekly after inoculation. Serum samples were taken at 3 and 6 weeks after tumours were detectable. Tumours were measured externally using vernier callipers (Figure S1A-D); according with local procedures, tumour volumes were calculated using the formula (A × b × b) being “A” the longest axis of the tumour and “b” the shortest. When the individual tumour sizes were over 1.5 cm diameter or overall tumour volume per mouse was over 10% of body weight, animals were killed by exsanguination and cervical dislocation as confirmation of death. Tumours were collected for histopathology and molecular biology assays. Immunohistochemistry stains included: CD31, alpha smooth muscle actin (aSMA), oestrogen receptor (OR), vascular endothelial growth factor (VEGF), carbonic anhydrase IX (CA-IX), periodic acid-Schiff (PAS), arginase and inducible nitric oxide synthase (iNOS) as detailed in Supplementary Information. Western blot for nitrotyrosine and VE-cadherin was performed according to standard methods, as was measurement of oxidative modification (Supplementary Information).

### Optoacoustic tomography

A commercial small animal multispectral optoacoustic tomography (MSOT) system (inVision 256-TF; iThera Medical GmbH) was used in this study. The system has been described in detail elsewhere.^30,31^ Briefly, a tunable (660–1300 nm) optical parametric oscillator (OPO), pumped by a nanosecond (ns) pulsed Nd:YAG laser, with 10 Hz repetition rate and up to 7 ns pulse duration is used for signal excitation. Light is delivered to the sample through a custom optical fibre assembly to obtain a uniform diffuse ring of illumination over the imaging plane. Coupling of the sample to the transducers is achieved using a water bath, filled with degassed and deionized water. An array of transducers covering an angle of 270° is used as the detector allowing tomographic reconstruction.

Mice were prepared according to our standard operating procedure^32^ and following UKCCCR guidelines.^28^ Briefly, mice were anaesthetised using <3% isoflurane in 100% oxygen and placed in a custom animal holder (iThera Medical), wrapped in a thin polyethylene membrane, with ultrasound gel (Aquasonic Clear, Parker Labs) used to couple the skin to the membrane. The holder was then placed within the MSOT system and immersed in degassed water maintained at 36 °C for Hb and HbO_2_ imaging acquisition. The animal respiratory rate was maintained in the range 70–80 b.p.m. with ~1.5% isoflurane concentration for the entire scan. The animal holder was translated along the oral-caudal axis of the tumour and serial images every 0.5 mm were taken for all the animals. Images were acquired using six wavelengths between 700 and 950 nm, with an average of 10 pulses per wavelength. Each slice took 7 s to acquire, with overall imaging sessions lasting for a time ranging between 3 and 8 min.

### Image and statistical analysis

All analysis was performed unblinded. Histopathological analysis of paraffin embedded tissue sections was performed on images scanned at ×20 magnification using an Aperio ScanScope (Leica Biosystem) scanner, a whole-tumour section of the wider tumour area was analysed. ROIs were drawn over the whole-viable tumour area. The percentage of viable area was estimated in H&E sections (Figure S1E). The parameters measured were the following: CD31 staining = positive pixel count/ROI area; CD31 microvessel density = vessels marked by CD31/ROI area; ASMA positivity = positive pixel count /ROI area; Mast cell density = cells marked by toluidine blue/ROI area, CA-IX staining = positive pixel count/ROI area. Oesotrogen receptor status was confirmed in MCF-7 tumours using the percentage of positive nuclei (Figure S1F). CD31/PAS analyses were performed as follows, for same slide analysis three random fields per slide were studied, blood vessels were identified and percentage of CD31 positive blood vessels were identified by an expert. The quantification was performed blindly. In sequential adjacent tissue sections. Slides were scanned in an Axio Scan Z1 (ZEISS) and Halo Software (v2.1.1602) was used to synchronise the images. PAS positive blood vessels were identified by a histopathologist (S.J.A.) by drawing ROIs in five random fields per tumour sample (magnification ×20). An algorithm to quantify corresponding CD31 intensity analysed the presence of the protein in the blood vessel area.

For arginase and iNOS IHC quantification was performed using Image J software. Colour deconvolution for Haematoxylin counterstain of DAB (H-DAB) coordinates was applied; after 8-bit conversion the same threshold (122 and 107 for iNOS and arginase, respectively) was applied to all the images. The positive pixels were counted using particle analysis. The average of the sum of the positive particles per field was the metric used for each sample and at least 3 fields (×20) were analysed per sample.

**Fig. 1:**
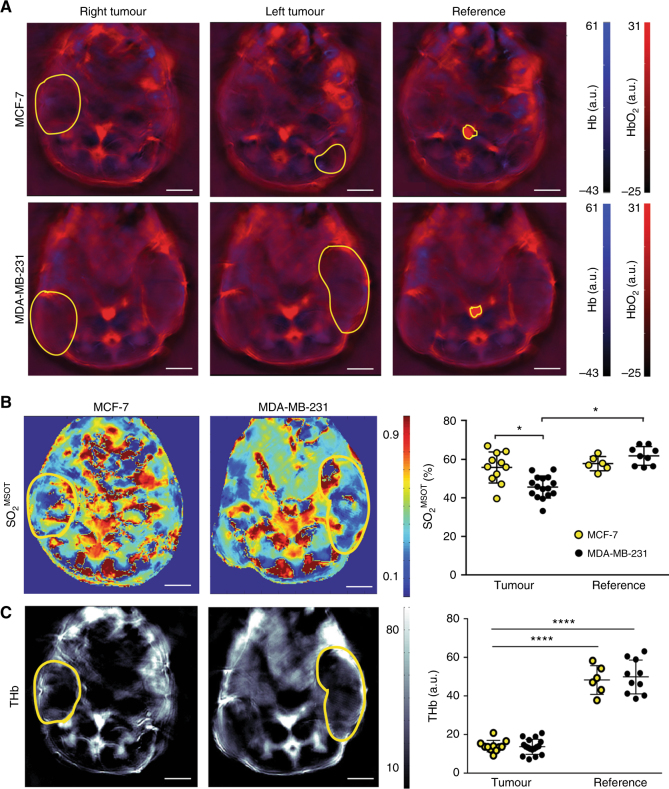
Optoacoustic tomography reveals oestrogen-independent MDA-MB-231 tumours have poorer oxygenation than oestrogen-dependent MCF-7 tumours and healthy tissue. **a** Regions of interest (yellow outline) were drawn in the optoacoustic tomography slice at which the tumour area burden was highest. A region containing the aorta and inferior vena cava was used as a baseline reference in normal tissue. Representative images show the spatial distribution of **b** oxygenation (SO_2_^MSOT^) and (**c**) total haemoglobin (THb) with tumour regions of interest overlaid (yellow outline). Quantification graphs are shown on the right, data were extracted from all regions of interest, and show a significantly higher oxygenation in MCF-7 compared to MDA-MB-231 (**b**) tumours and a decrease in THb (**c**). *n*^MCF−7^ = 11; *n*^231^ = 16, data expressed as mean ± SEM. *p < 0.05, ****p < 0.0001. Statistical significance was assessed by paired two-tailed *t*-test within a single-tumour type and by unpaired two-tailed *t*-test between tumour types

OT analysis was performed using ViewMSOT software (v3.6.0.119; iThera Medical GmbH). Model-based image reconstruction and multispectral processing were applied to retrieve the relative signal contributions of oxy- (HbO_2_) and deoxy- (Hb) haemoglobin. Region of interests (ROIs) were drawn for the tomographic section in which the tumour presented the largest area (Fig. 1a). Reference values from an ROI drawn around the abdominal aorta and vena cava were taken in the same anatomical plane, before they branch for the junction with the iliac bone (Fig. 1a). ROIs were drawn over: the whole tumour; tumour rim (taken as 1 mm outer circumference of the tumour) and tumour core (the tumour area inside the 1 mm rim). OT measured ROI tumour areas correlated with tumour volume in both models (Figure S1G,H). Average and maximum intensities for HbO_2_ and Hb were measured. OT is only able to accurately resolve absolute SO_2_ if the recorded signal is directly related to the absorbed optical energy distribution, which requires knowledge of the light fluence distribution, system response and Grueneisen parameter.^33^ We therefore denote the oxygenation metric derived in this study as an apparent metric, SO_2_^MSOT^ rather than absolute SO_2_. SO_2_^MSOT^ was computed as the ratio of HbO_2_ to total haemoglobin signal in the ROI (THb = HbO_2_ + Hb).

Statistical analysis was performed using Prism (GraphPad). Each tumour was considered as an independent biological replicate. All data are shown as mean ± SEM unless otherwise stated. For end time point comparisons between the two cohorts for optoacoustic signals, histopathology results, plasma quantification, ELISA and western blot, unpaired two-tailed *t*-test was performed. For end time point comparisons between tumour rim and core within the same cohort, paired two-tailed *t*-test was performed. For time course comparison within the same cohort, one-way ANOVA followed by Tukey’s test for multiple comparisons when applicable and Pearson correlation was tested. Significance is assigned for *p*-values <0.05.

## Results

### Non-invasive assessment of tumour vasculature using optoacoustic tomography reveals different phenotypes between MCF-7 and MDA-MB-231 models

The two breast cancer cell lines were chosen due to their biological differences: less aggressive, better differentiated phenotypes usually present a pronounced paracrine activity and recruit a more complex microenvironment, while the more aggressive, less differentiated types tend to acquire mesenchymal characteristics.^34^ Therefore, we expected these two cell lines present differences in vasculature when orthotopically implanted in mice.

Exemplar OT image slices are shown in Fig. 1a. Reproducibility based on the coefficient of variation for all OT metrics was tested over three repeated measurements made over 48 h (Figure S2A). As we assessed previously in healthy organs, oxygen saturation (SO_2_^MSOT^) and total haemoglobin (THb) exhibit a lower coefficient of variation compared to the direct measurements of oxy- and deoxy-haemoglobin (Hb and HbO_2_),^32^ hence we focused on the measurement of SO_2_^MSOT^ and THb for the remainder of the analysis. The SO_2_^MSOT^ in established MDA-MB-231 tumours was significantly lower than both MCF-7 tumours and the reference region (Fig. 1b; SO_2_^MSOT^ = 46 ± 1% vs. 55 ± 2 and 55 ± 3% for the reference region in the same mice, respectively). The SO_2_^MSOT^ in MCF-7 tumours were equivalent to the reference region in the same mice. The THb in the tumour is significantly lower than in the reference and no difference was observed between the tumours generated by both models (Fig. 1c).

Since OT is fast, non-invasive and label free, longitudinal monitoring of tumour development is possible. We performed weekly imaging sessions in all mice following tumour inoculation. The different oxygenation levels observed in the established tumours is also discernible early in the time course analysis and maintains throughout tumour growth. A trend towards decreasing SO_2_^MSOT^ (Figure S2B) is confirmed when the SO_2_^MSOT^ of the MCF-7 tumour is corrected by the SO_2_^MSOT^ of the reference (Figure S2C, Pearson *p* = 0.001), accounting for any drifts in OT performance over longer term studies. THb is not affected during tumour growth (Figure S2E). No change in SO_2_^MSOT^ or THb is observed in the MDA-MB-231 tumours throughout the time course.

### Optoacoustic tomography detects spatial variation in vascular maturity

**Fig. 2:**
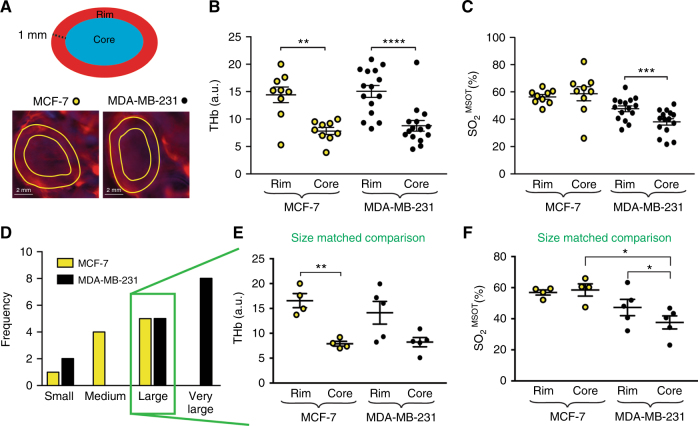
Optoacoustic tomography provides a non-invasive assessment of the rim–core vascular phenotypes of both breast cancer models. **a** Rim data were taken from a region of interest drawn around the outside of the tumour, then shrunk by a radial distance of 1 mm. Significant differences in THb and SO_2_^MSOT^ were seen between the rim and core of MDA-MB-231 tumours, though only THb showed a rim–core variation in MCF-7 tumours **b**, **c**. Extracting “large” (20–30 mm^2^ OT area) tumours for a size matched analysis (**d**) showed similar THb values but different SO_2_^MSOT^ between rims and cores **e**, **f**. Statistical significance was assessed by paired two-tailed *t*-test within a single-tumour type and by unpaired two-tailed *t*-test between tumour types. For (**b**) and (**c**) *n*^MCF−7^ = 11; *n*^231^ = 15, for (**e**) and (**f**) *n*^MCF−7^ and *n*^231^ = 4. All panels data are expressed as mean ± SEM. *p < 0.05, **p < 0.01, ***p < 0.001, ****p < 0.0001

Tumour oxygenation generally decreases with depth, as the outer rim of the tumour can be well oxygenated by diffusion but the core of the tumour experiences perfusion- and diffusion-limited access to oxygen.^35^ We thus hypothesised that OT would detect a lower total haemoglobin and oxygen saturation in the core of our tumour models. To test this hypothesis, we drew additional regions of interest to delineate two areas of the tumour (Fig. 2a): the rim, defined as the perimeter area of 1 mm depth into the tumour; and the core, the rest of the tumour area after the rim has been excluded. Both tumour models present a significant reduction in THb between the rim and the core (Fig. 2b; MCF-7 THb = 14.4 ± 1.4 rim vs. 7.8 ± 0.6 core; MDA-MB-231 THb = 15.0 ± 1.1 rim vs. 8.7 ± 0.9 core). However, only MDA-MB-231 tumours show a significant reduction in SO_2_^MSOT^ between the rim and the core (Fig. 2c; MDA-MB-231 SO_2_^MSOT^ = 47.7 ± 2.1 rim vs. 38.0 ± 2.3 core). The underlying Hb and HbO_2_ values (Figure S3A,B) show comparable trends.

Given the different growth rates observed for the two breast tumour models (Figure S1A-D), we also performed a size-matched analysis of this spatial heterogeneity. We divided the tumours into small (<10 mm^2^), medium (10–20 mm^2^), large (20–30 mm^2^) and very large (>30 mm^2^) size classes according to ROI area measured by OT (Fig. 2d), which correlates with tumour volume (Figure S1G,H). Equal numbers of “large” MCF-7 and MDA-MB-231 tumours were available, so we chose this group for our size-matched analysis. The rim–core behaviour found within each model in the whole cohort analysis persisted in the size-matched analysis (Fig. 2e, f). When comparing the THb between models (i.e., MCF-7 rim compared to MDA-MB-231 rim; MCF-7 core compared to MDA-MB-231 core) we observed no significant differences (Fig. 2e). The SO_2_^MSOT^ was similar for the rims of both groups while the core of MCF-7 had significantly higher SO_2_^MSOT^ than the core of MDA-MB-231 tumours of similar size (Fig. 2f). Again, the underlying Hb and HbO_2_ values (Figure S3C,D) confirm these findings. The dynamic of the oxygenation in rim and core was also analysed over time (Figure S3E). The behaviour was similar to previous result, with the average oxygenation being higher in MCF-7 than in MDA-MB-231. The levels of oxygenation in MCF-7 are higher in the core at the earlier time points but the rim and core become comparable at later time points. MDA-MB-231 tumours showed similar oxygenation in the rim compared to the core in the first stages of tumour development, with disparity arising later. Taken together, these findings show MCF-7 tumours exhibit high oxygenation, similar to healthy tissue, indicating a functional vasculature in both their rim and core; conversely, MDA-MB-231 tumours show a poorer oxygenation overall, likely driven by their poorly oxygenated core.

### Histological assessment proves the higher maturity of the vessels in MCF-7 tumours

**Fig. 3:**
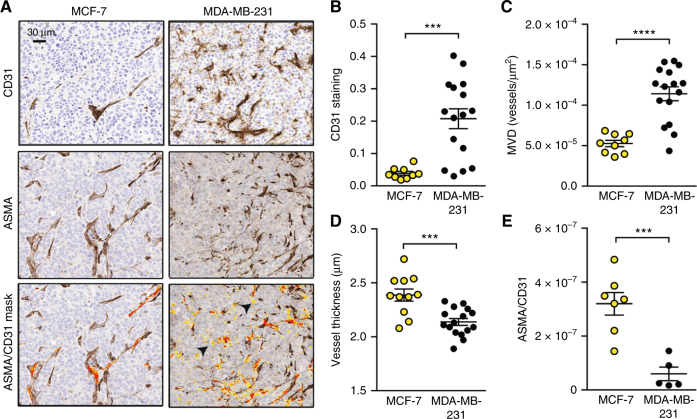
MDA-MB-231 exhibit a high-microvessel density but relatively poor maturity compared to MCF-7. **a** IHC representative micrographs for each tumour type stained with CD31 to mark endothelial cells and ASMA to mark the supporting pericyte layer (ASMA+ cells surrounding blood vessels). The lowest panel shows the mask used to count co-localised ASMA and CD31 staining on adjacent sections (orange overlap/yellow CD31+ only). Arrowheads indicate CD31+ with no ASMA staining in MDA-MB-231. Scale bar = 30 µm. MDA-MB-231 tumours show increased overall CD31 staining (**b**) and microvessel density (MVD, **c**), but decreased vessel thickness (**d**) and ASMA coverage (**e**) compared to MCF-7. For **b**, **c** and **d**, *n*^MCF−7^ = 12 and *n*^231^ = 16. For E, *n*^MCF−7^ = 7 and *n*^231^ = 4. All panels, data expressed as mean ± SEM. ***p < 0.001, ****p < 0.0001 by unpaired two-tailed *t*-test

OT assessment of THb and SO_2_^MSOT^ relies on the presence of blood within the tumour mass; consequently, differences in vascular density and function would be expected to modify these imaging biomarkers. Mature, functional blood vessels are supported by an effective vascular network that includes at least two cell types: endothelial cells, forming the blood vessel wall; and pericytes, providing structural coverage to resist the blood flow. In order to establish the underlying vascular density and function in the two breast tumour models, we examined the endothelial and pericyte layers using IHC for CD31 and α-Smooth Muscle Actin (ASMA), respectively (Fig. 3a). Both overall CD31 staining and microvessel density (MVD) were significantly higher in MDA-MB-231 tumours (Fig. 3b, c; CD31: 0.04 ± 0.006 MCF-7 vs. 0.21 ± 0.03 MDA-MB-231; MVD: 5.3 × 10^−5^ ± 0.4 × 10^−5^ MCF-7 vs. 1.1 × 10^−4^ ± 0.7 × 10^−5^ MDA-MB-231). The vessel wall thickness was significantly higher in MCF-7 tumours (Fig. 3d; 2.38 ± 0.05 MCF-7 vs. 2.14 ± 0.03 MDA-MB-231), which also showed a much higher staining of ASMA co-localised with CD31 (Fig. 3e; 3.4 × 10^−7^ ± 1.1 × 10^−7^ MCF-7 vs. 0.6 × 10^−7^ ± 0.2 × 10^−7^ MDA-MB-231). While MCF-7 tumours exhibit mostly strong areas stained by CD31 and always surrounded by strong ASMA staining, MDA-MD-231 tumours showed positive low staining of CD31 in areas where no ASMA staining was detected, probably representing the immature vasculature generated in this tumour model (Fig. 3a, arrowheads). These results indicate that the blood vessels in MCF-7 tumours present pericyte or media layer, being more mature compared to MDA-MB-231 tumours, which contain poorly developed blood vessels.

### The angiogenic-related microenvironment is different between both models

**Fig. 4:**
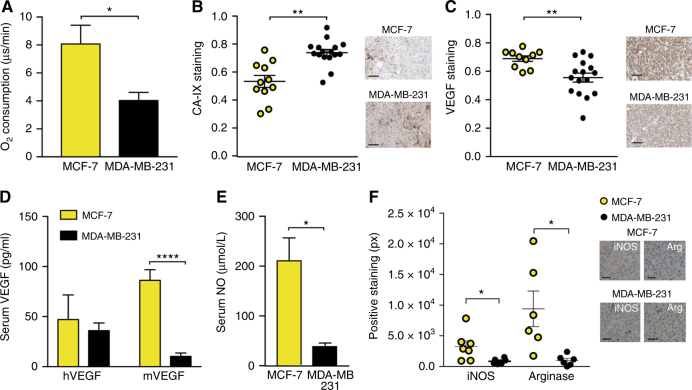
The hypoxic and inflammatory phenotype differs between the two breast tumour models. MDA-MB-231 cells have a lower oxygen consumption rate (**a**) yet show higher hypoxia in tumours (**b**). VEGF staining is lower in MDA-MB-231 tumours (**c**). Serum mouse VEGF (mVEGF, **d**) and nitric oxide (NO, **e**) are also lower in MDA-MB-231 tumours. **f** MCF-7 shows an inflammatory phenotype, with higher staining for iNOS and Arginase indicating the presence of type 1 and 2 macrophages respectively. Scale bars in (**b**) and (**c**) = 50 µm; **f** = 20 µm. For (**b**) and (**c**) *n*^MCF−7^ = 12 and *n*^231^ = 16. For (**d**) and (**e**) *n*^MCF−7^ = 6 and *n*^231^ = 9. All panels, data expressed as mean ± SEM, *p < 0.05, **p < 0.01, ***p < 0.001, ****p < 0.0001 by unpaired two-tailed *t*-test

In order to characterise the origin of the different vasculature we studied different aspects that are able to influence the angiogenesis process. We sought to better understand the connection between the non-invasive OT imaging data and the underlying tumour biology relating to vessel formation, including hypoxia and inflammation. The relationship between blood oxygenation as measured by OT, and tissue oxygenation is affected by the vascular structure and metabolism of the tumour. Therefore, we first assessed the basal oxygen consumption of both cell lines, finding that the oxygen consumption in MCF-7 cells is significantly higher than in MDA-MB-231 cells (Fig. 4a; 8.07 ± 1.33 MCF-7 vs. 4.03 ± 0.58 MDA-MB-231). We then confirmed that MDA-MB-231 tumours also exhibit higher levels of hypoxia, by assessing tumour expression of Carbon Anhydrase IX (CA-IX), a protein upregulated in hypoxic conditions (Fig. 4b; 0.53 ± 0.04 MCF-7 vs. 0.73 ± 0.02 MDA-MB-231). This reinforces our vascular IHC analysis, indicating that the lower levels of blood oxygenation in the MDA-MB-231 tumours are likely due to the lower vascular maturity, which limits oxygen delivery to the tumour tissue.

Next, we determined the tumour and serum levels of vascular endothelial growth factor (VEGF), one of the most important pro-angiogenic factors.^36^ VEGF can be secreted by stromal cells, due to inflammation, and directly by tumour cells under hypoxia. The local levels of VEGF in the tumour assessed by IHC were significantly higher in MCF-7 than in MDA-MB-231 tumours (Fig. 4c; 0.63 ± 0.06 MCF-7 vs. 0.55 ± 0.03 MDA-MB-231). To elucidate the source of VEGF, we measured the serum levels of human VEGF (hVEGF), coming from the tumour cells, and mouse VEGF (mVEGF), coming from host tissue. Although the levels of hVEGF are similar in both tumour models, mVEGF is significantly increased in the serum of mice bearing MCF-7 (Fig. 4d; 86.2 ± 10.6 MCF-7 vs. 15.7 ± 6.2 MDA-MB-231), pointing to the stromal compartment as the main source of VEGF in MCF-7 model. Interestingly, another important mediator of inflammation and endothelial homoeostasis, nitric oxide (NO), was also significantly increased in serum of mice bearing MCF-7 tumours (Fig. 4e; 210.6 ± 45.9 MCF-7 vs. 40.8 ± 4.8 MDA-MB-231). Since NO can be produced by macrophages, we assessed by IHC iNOS expression, to denote type 1 macrophages and arginase expression, to denote type 2 macrophages.^37^ The levels of these proteins (both of mouse origin) were significantly higher in MCF-7 tumours (Fig. 4f; iNOS: 3284 ± 888 MCF-7 vs. 850 ± 182 MDA-MB-231; arginase: 10,111 ± 2542 MCF-7 vs. 1002 ± 298 MDA-MB-231). This increase occurs without any coincident increase in oxidative stress (Figure S4). Taken together these results suggest that the main driving force for angiogenesis in MCF-7 tumours is inflammation rather than local hypoxic stimuli.

**Fig. 5:**
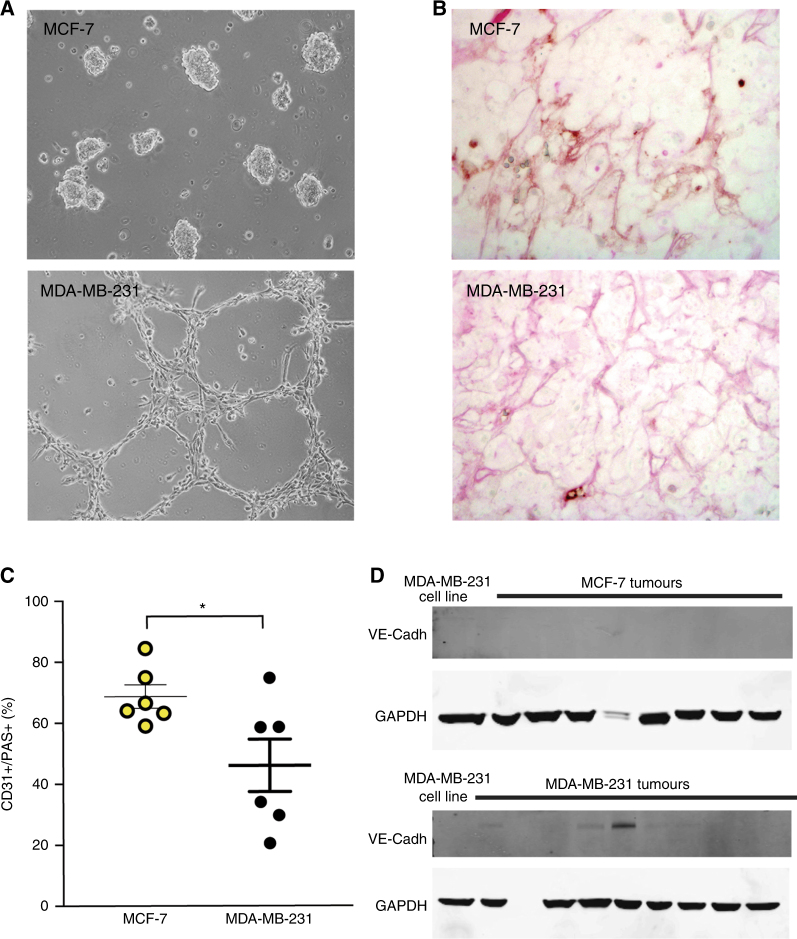
Assessment of vascular mimicry. **a** Representative micrographs (magnification ×20) of tubular-like structures generated in matrigel 3D in vitro culture for MDA-MB-231 cells; no such structures are observed in MCF-7 cells. This phenotype is associated with vascular mimicry. **b** Representative micrographs (magnification ×40) of tumour sections stained with PAS and CD31. **c** All PAS-positive blood vessels were identified and the CD31 positivity of these blood vessels was then evaluated. The number of CD31+/PAS+ blood vessels was significantly lower in MDA-MB-231 compared to MCF-7 tumours. **d** Western blot for protein levels of VE-Cadherin in MCF-7 and MDA-MB-231 xenograft tumours ex vivo provided confirmation of these in vitro findings. GAPDH is shown as a house-keeping protein. **b**, **c** *n*^MCF−7^ = 6 and *n*^231^ = 6 data expressed as mean ± SEM, *p < 0.05 by unpaired two-tailed *t*-test. **d** *n*^MCF−7^ = 8 and *n*^231^ = 15

Given the lack of macrophage infiltration and low levels of mVEGF, an inflammatory stimulus could not explain the vascular features of the MDA-MB-231 tumours. Furthermore, despite a high density of tumour blood vessels, MDA-MB-231 tumours show significantly lower VEGF staining. To clarify which vascular pathway was activated in these cells, we checked the ability of these cells to trans-differentiate into endothelial-like tubular structures in matrigel, a process referred to as ‘vascular mimicry’ (VM), which has been previously reported. In line with previous results, our MDA-MB-231 cells form such structures after 24 h of 3D matrigel culture (Fig. 5a). We analysed the in vivo capacity of VM in both cell lines by studying the expression of CD31 and PAS in blood vessels. Vessels derived by VM are PAS positive but do not express CD31.^38^ We identified PAS-positive blood vessels and then quantified their positivity for CD31 staining (Fig. 5b; see also Figure S5). We observed significantly fewer CD31+/PAS+ vessels in MDA-MB-231 tumours compared with MCF-7 derived tumours (Fig. 5c). The MDA-MB-231 tumours also showed detectable expression of VM marker VE-Cadherin^39^ (Fig. 5d; positive in 8 of 15 tumours compared to 0 of 8 tumours in MCF-7). The presence of vascular mimicry that we have identified in MDA-MB-231 tumours may explain in part their poorer overall oxygenation.

## Discussion

The aim of this study was to define whether OT can detect differences in the vasculature and oxygenation of two biologically different breast cancer xenografts. We sought to determine whether the features defined by OT were connected with the underlying vascular phenotype. To achieve these aims, we used two different breast cancer cell lines exemplifying different stages of the breast cancer evolution. We studied the functional OT image information relating to the vasculature, including changes in haemoglobin concentration (THb) and oxygenation (SO_2_^MSOT^) and evaluated the biological features that underlie these features.

Globally, our results confirm OT is sufficiently sensitive to differentiate in vivo and in real time the vasculature features of the tumours generated by two breast cancer cell lines as representing different stages of the disease. Our oestrogen-dependent MCF-7 tumours showed an average oxygenation similar to normal blood vasculature, which was also much higher than MDA-MB-231 tumours. The average oxygenation of MCF-7 tumour tissue decreased over time as the tumour developed, whereas, in MDA-MB-231 tumours, oxygenation remained low across the time course. The work from Wilson et al.^40^ described alterations in THb and increased SO_2_ during initiation, promotion and progression in a transgenic breast cancer model. They also described a reduction in basal systemic haemoglobin levels in the mice bearing invasive tumours compared to the normal and hyperplasia bearing mice. Increases in SO_2_ and THb that were observed during hyperplasia and the malignant transformation are not recapitulated in our model, but consistent with our results they describe a decrease SO_2_ and THb during the in situ carcinoma and invasive disease. In addition, their ex vivo analysis actually indicated increased vessel density in the invasive tumours, as we show here for the more aggressive MDA-MB-231 tumours compared to MCF-7.

Both tumour types showed a pronounced rim–core effect, with more haemoglobin present in the rim of the tumours. MCF-7 tumours were similarly oxygenated in both the rim and core; whereas, MDA-MB-231 tumours had a poorly oxygenated tumour core. The rim–core effect in OT and histopathology data for MDA-MB-231 observed here was consistent with previous work by Bar-Zion et al.,^41^ where higher THb and SO_2_ values were shown in tumour rims.

Comparing the vascular phenotype of these tumour models using immunohistochemistry, we found that while MDA-MB-231 tumours had a far higher microvessel density, but their vessels were poorly developed, with little pericyte coverage. These findings explain our OT image features, particularly the low oxygenation measured in MDA-MB-231 tumours, as vessels with low-pericyte coverage lack vasoactivity and increased pericyte coverage has previously been linked to tumour oxygenation.^42^ Furthermore, the similar OT haemoglobin signal between the tumour types indicates many of the vessels present in MDA-MB-231 tumours are likely to be non-functional.

To understand the origins of the differing vascular phenotypes of these tumours, we examined their hypoxic and inflammatory phenotypes. The biochemical pattern shown by MCF-7 tumours, with high levels of VEGF and NO and positive expression of proteins involved in macrophage function, indicates activation of the angiogenic pathway and also the involvement of inflammation and/or endothelial cells. Conversely, MDA-MB-231 tumours appear less dependent on angiogenesis, with expression of human VE-cadherin measured in tumour extracts. They are also characterised by PAS+ blood vessels negative for CD31 expression, indicating vascular mimicry. Such features of vasculogenesis in these cell lines have previously been described in vitro^43^ and our in vivo results in tumour tissue support their findings. However, the presence of a large number of CD31+ vessels in our MDA-MB-231 tumours may be considered unusual, as previous work suggests that vessels formed by vascular mimicry do not express this endothelial marker.^44^ The presence of CD31+ vessels could be due to the co-existence of both vascular mimicry and angiogenesis, or due to vascular mimicry induced by macrophages, in which the presence of CD31+ cells has been recently described.^45^ Nonetheless, the deficient structure of vessels arising from vascular mimicry would impair exchange of oxygen from haemoglobin, which may provide part of the explanation for differences in oxygenation measured with OT. To our knowledge, this is the first time that OT has been used in tumours presenting features of vascular mimicry and reinforces the potential application of OT to monitor vasculogenic processes in tumours.^46,47^ Nonetheless, further experiments have to be performed to confirm whether OT can be more broadly applied to discriminate vascular mimicry and angiogenesis.

There remain some key limitations to our study. First, we examine an orthotopic xenograft model system using only two cell lines, representing oestrogen-dependent and independent disease but there are other subtypes that are relevant for the disease. The absence of an adaptive immune system in the nude mice used for the study may also impact tumour development and the resulting vascular phenotype. Our findings should therefore be further verified in additional models, including transgenic or patient-derived tumour models, prior to being evaluated in a clinical setting. Second, we analyse only relative values of haemoglobin concentration and oxygenation, rather than absolute values. To derive absolute values would require compensation for differential attenuation of the full range of wavelengths used via light fluence correction of the OT data; application of light fluence correlation in vivo is the subject of current research^48^ but is not yet validated for routine use. As a result, only relative values were presented here.

Clinical applications of OT in breast cancer appear promising compared to existing clinical imaging modalities, particularly when applied to dense breasts.^22,23^ Limitations with sensitivity and specificity remain, which may be influenced by the type of detector (hand held, frame with compressing plates or cup shape) and the available wavelengths for imaging. Here we confirmed that OT image data acquired in the wavelength range from 700 to 950 nm to determine blood concentration and oxygenation reflects the underlying vascular phenotype of two breast tumour models, moving OT one step closer to validation as an imaging modality in breast cancer. Future studies will be needed to elucidate whether OT meets the clinical need for an accurate, fast and affordable tool to provide validated imaging biomarkers of tumour angiogenesis.

## Electronic supplementary material

Supplementary Information
