# Supplementary material for: Optoacoustics delineates murine breast cancer models displaying angiogenesis and vascular mimicry
Source: Br J Cancer. 2018 Mar 26;118(8):1098–106. doi: 10.1038/s41416-018-0033-x (PMC5931091; doi:10.1038/s41416-018-0033-x)

Supplementary Information for:

Optoacoustics delineates murine breast cancer models displaying angiogenesis and vascular mimicry

Isabel Quiros-Gonzalez, Michal R Tomaszewski, Sarah Aitken, Laura Ansel-Bollepalli, Leigh-Ann McDuffus, Michael Gill, Lina Hacker, Joanna Brunker and Sarah E. Bohndiek.

Contents

Supplementary Methods	p.2
Supplementary Figure 1	p.6
Supplementary Figure 2	p.8
Supplementary Figure 3	p.10
Supplementary Figure 4	p.12
Supplementary Figure 5	p.13

Supplementary Methods

Cell lines

In vitro experiments were performed when cells were between passage 20-25 for both MCF-7 and MDA-MB-231. Authentication using Genemapper ID v3.2.1 (Genetica) by STR Genotyping (1/2015) showed 100% match with the reference sequence in both cases. Cells were maintained in DMEM supplemented by 10% of FBS at 37ºC in 5% CO_2. Oxygen consumption was measured using the MitoXpress Xtra Oxygen Consumption assay.

MitoXpress Xtra Oxygen Consumption assay

Oxygen consumption of the MCF-7 and MDA-MB-231 cells was assessed by the *MitoXpress Xtra Oxygen Consumption (HS Method)* assay (LuxCel Bioscience). Cells were seeded into a 96-well plate in 2 concentrations (50,000 and 100,000 cells/well, n=15) in 150 µL of complete media. The next day MitoXpress Xtra was added to each well in fresh media following manufacturer instructions. Two drops of HS mineral oil were added to each well to prevent oxygen exchange. Positive and negative controls included 1 mg/mL of Glucose Oxidase, 1 µL of 150 µM Carbonyl cyanide-4-(trifluoromethoxy)phenylhydrazone (FCCP) (Sigma Aldrich) solution and 1 µL of 150 µM Antimycin A respectively. Measures were taken at 37°C for 2.5 h in a plate reader (CLARIOstar, BMG Labtech) according to a protocol described elsewhere (Hynes *et al*, 2013). The slopes of fluorescent lifetime changes were extracted for each cell line and cell density were extracted as a quantification of the oxygen consumption in the well.

A duplicate plate was used to quantify the relative cell densities in the wells. The plate without media was frozen at -80°C. After thawing 50 µL of TNE buffer (Tris 50 mM, NaCl 100mM and EDTA 0.1 mM) buffer with 20 µg/mL Hoechst 33342 was added to each well and incubated for 15 minutes at room temperature. The plate was scanned at 361 nm excitation and 497 nm emission (CLARIOstar, BMG Labtech) and the fluorescence signal intensity was used as a measure of the cell density.

Measurements of oxidative modification

For blood nitration content, terminal blood collection was performed in heparinised tubes. Samples were centrifuged and diluted for quantification with Parameter™ Total Nitric Oxide (NO) and Nitrate/Nitrite Assay (R&D) following manufacturer's instructions. The detection is based on the colorimetric detection of nitrite by the Griess Reaction. Optical densities at 540 nm (signal) and 690 nm (correction) were detected and the relative concentration of NO was extrapolated from a standard curve.

For detection of the redox modified nucleotide 8-hydroxy-2'deoxyguanosine, we purified the DNA from paraffin embedded tumour tissue and detected the modification by ELISA. Briefly, DNA from 3-5 sections (5μm thickness) were extracted by using the PaxGene DNA kit (Qiagen) following the manufacturer's instructions. For all the tumours 2 μg of DNA were loaded into each well for the immunodetection using HT 8-oxo-dG ELISA kit II (Trevigen) following manufacturer's instructions.

Western-blot for nitrotyrosine and VE-cadherin

Tissue was homogenised and lysed with Pierce® RIPA buffer (Thermo Scientific) plus Halt protease and phosphatase inhibitors cocktail (Thermo Scientific). Protein concentration was determined by using the Direct Detect system (Millipore) and 45 μg of protein was loaded for electrophoresis. Proteins were dry-transferred to a PDVF membrane using iBLOT2.0 system. For nitrotyrosine, all tumour samples were compared with an extract from MDA-MB-231 cells treated with peroxynitrite 10 μM ($ONOO^-$) taken as a reference. Quantification was performed for the whole lane. For VE-Cadherin only presence- absence was assessed. GAPDH was used as house-keeping protein. Antibodies and conditions: Anti-Nitrotyrosine (Cayman Chemical) (1:300), anti-VE-Cadherin (1:1000) and anti-GAPDH (1:5000) (both Cell Signalling Technologies) were incubated overnight at 4 °C. Secondary antibodies were anti-Rabbit IRDye® 680LT (LI-COR) and anti-Mouse IRDye® 680LT (both 1:15000). Band intensity quantification was performed using Image J. For nitrotyrosine, all bands detectable from ~35 KDa to 100 KDa were measured and normalised against GAPDH. For VE-Cadherin, only the presence or absence of signal was counted.

Immunohistochemistry and special staining

Tissues were collected and fixed in 4% PFA for 24h. Samples were processed by the Cancer Research UK Cambridge Institute Histopathology Core. Tumour tissues were embedded in paraffin, sectioned and rehydrated. Immunohistohemistry for CD31, alpha Smooth Muscle Actin (aSMA), oestrogen receptor (OR), Vascular Endothelial Growth Factor (VEGF) and Carbonic Anhydrase IX (CA-IX) was performed in a BOND automated stainer (Leica Biosystems) at the following concentrations 1:50, 1:500, 1:200, 1:250 and 1:1000. All the antibodies needed from antigen retrieval. Antigen retrieval was performed previous to primary antibody incubation as follows: Proteinase K 10' at 37ºC for CD31, Tris-EDTA Heat shock for aSMA and Sodium Citrate heat shock for OE, VEGF and CA-IX. Toluidine blue for mast cells staining was performed following standard protocols.

Immunohistochemistry for Arginase and inducible Nitric Oxide Synthase (iNOS both Abcam) was performed manually. Briefly, after deparaffination and rehydration slides underwent antigen retrieval (10 mM Citrate buffer, 20 min, 95 °C), samples were blocked for 30 min using the Endogenous Blocking reagent (Dako). Primary antibodies anti-Arginase (1:200) and anti-iNOS (1:400) were diluted in 1% Cold Water Fish Skin gelatine in PBS and incubated for 40 minutes at room temperature. After serial washes (2x 5 min, PBS) the slides were incubated with the secondary anti-Rabbit+anti-Mouse HRP-conjugated solution (Dako) for 30 min. Di-amino-benzidine (DAB) was used as a substrate.

For Periodic Acid Schiff (PAS) staining alone, slides rehydrated were incubated in α-amilase for 30 min. After washing, slides were incubated in Acid Schiff for 5 min and subsequently in Schiff Reagent for another 15 min. Light Haematoxylin (40 s) was used as counterstain. Results are shown in Supplementary Figure 5. For Periodic Acid Schiff (PAS) staining with CD31, slides rehydrated were incubated in were incubated in Acid Schiff for 5 min and subsequently in Schiff Reagent for another 15 min. Light Haematoxylin (40 s) was used as counterstain. When indicated, after PAS staining slides were treated with Proteinase K at 37 °C for 10 minutes and blocked with the endogenous blocking solution

(Dako) and incubated with CD31 (1:100). The secondary anti-Rabbit+anti-Mouse HRP-conjugated solution (Dako) was incubated for 30 min. Di-amino-benzidine (DAB) was used as a substrate.

Mouse and human VEGF inmunodetection in serum

For mouse and human VEGF immunodetection (mVEGF and hVEGF respectively) Quantikine® ELISA kits (R&D) were used. Plasma samples were diluted 1:10 and 50 µl were used to quantify the concentration added per well. The procedure was carried out following manufacturers instructions. The colorimetric reaction was quantified at 450 nm. The final concentrations were extrapolated by correlation of the log of the O.D. with the log of the values in the standard curve.

Supplementary Figures

Figure S1: Proliferation characteristics of MCF-7 and MDA-MB-231. (A) MCF-7 tumours were studied up to 7 weeks post inoculation, after which mice had to be sacrificed due to side effects from the implanted oestrogen pellet. (B) MDA-MB-231 tumours were studied up to 12 weeks post inoculation, after which mice were sacrificed due to ethical animal welfare limits. (C, D) Individual growth curves for both MCF-7 and MDA-MB-231 tumours. (E) H&E sections were used to identify the necrotic areas; a magnification of a field showing representative necrotic areas is shown (left) together with a quantification of the necrotic area (right). (F) Oestrogen receptor (OR) status was confirmed in MCF-7 tumours, with negligible oestrogen positive nuclei observed in MDA-MB-231 assessed by unpaired t-test. Assessment of tumour volume by calipers (as shown in A-D) was compared to assessment using the largest observable tumour area within a single optoacoustic tomography (OT) slice and showed a significant positive Pearson correlation for MCF-7 (G) and MDA-MB-231 (H). For (A-D) $n^{MCF\text{-}7}$ =11, for (E,F) $n^{MCF\text{-}7}$=12. All panels n^{231}=16, data expressed as mean±SEM. ** $p<0.01$, *** $p<0.001$, **** $p<0.0001$.

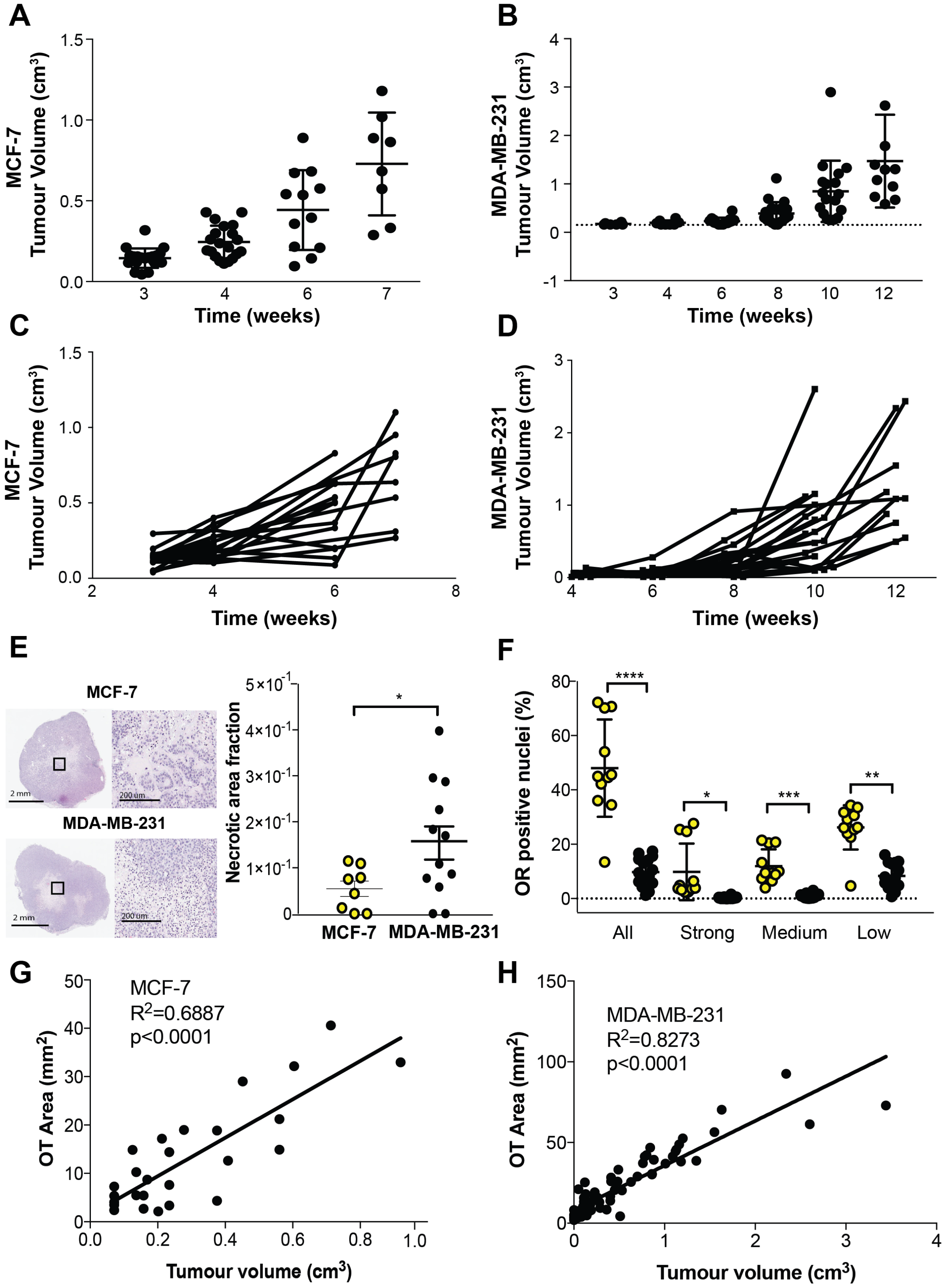
A
MCF-7
Tumour Volume (cm³)
Time (weeks)
B
MDA-MB-231
Tumour Volume (cm³)
Time (weeks)
C
MCF-7
Tumour Volume (cm³)
Time (weeks)
D
MDA-MB-231
Tumour Volume (cm³)
Time (weeks)
E
MCF-7
MDA-MB-231
Necrotic area fraction
MCF-7
MDA-MB-231
F
OR positive nuclei (%)
All
Strong
Medium
Low
G
MCF-7
R²=0.6887
p<0.0001
OT Area (mm²)
Tumour volume (cm³)
H
MDA-MB-231
R²=0.8273
p<0.0001
OT Area (mm²)
Tumour volume (cm³)

Figure S2: Optoacoustic tomography allows longitudinal monitoring of oxygenation and total haemoglobin. (A) The coefficient of variation (COV) was assessed for measurements of oxygen saturation (SO_2^{MSOT}), total haemoglobin (THb), deoxyhaemoglobin (Hb) and oxyhaemoglobn (HbO_2). Data were extracted from regions of interest placed over MCF-7 and MDA-MB-231 tumours, as well as from the reference area (Ref), in mice imaged repeatedly at 3 time points (0h, 24h, and 48h). COV values for MDA-MB-231 tumours are: 6.5±2.8% (SO_2^{MSOT}), 28.3±25.2% (THb), 51.7±22.6% (Hb) and 39.5±23.1% (HbO_2). COV values for MCF-7 tumours are: 5.0±1.6% (SO_2^{MSOT}), 11.4±0.9% (THb), 17.8±4.5% (Hb) and 15.5±3.5% (HbO_2). COV values for the reference are: 3.1±1.8% (SO_2^{MSOT}), 10.1±3.4% (THb), 21.6±13.6% (Hb) and 8.8±3.9% (HbO_2). (B) SO_2^{MSOT} shown over the time course of the experiment. SO_2^{MSOT} remains approximately constant in the MDA-MB-231 tumour region over time from study initiation, whereas a trend towards decreasing oxygenation is observed in MCF-7. (C) These trends remain when the oxygenation signal from the tumour (tum) is normalised to that extracted from the reference region around the aorta and inferior vena cava (ref). Normalisation was performed to ensure that any changes in systemic blood oxygenation were not affecting our results. (D) No significant change THb was observed across the time course. All panels data expressed as mean±SEM. * $p<0.05$, ** $p<0.01$, *** $p<0.001$, **** $p<0.0001$. (A) $n^{MCF\text{-}7}= 3$, $n^{231} = 4$ and $n^{reference} = 3$. (B) (C) and (D) $n^{MCF\text{-}7} = 11$ and $n^{231} = 16$.

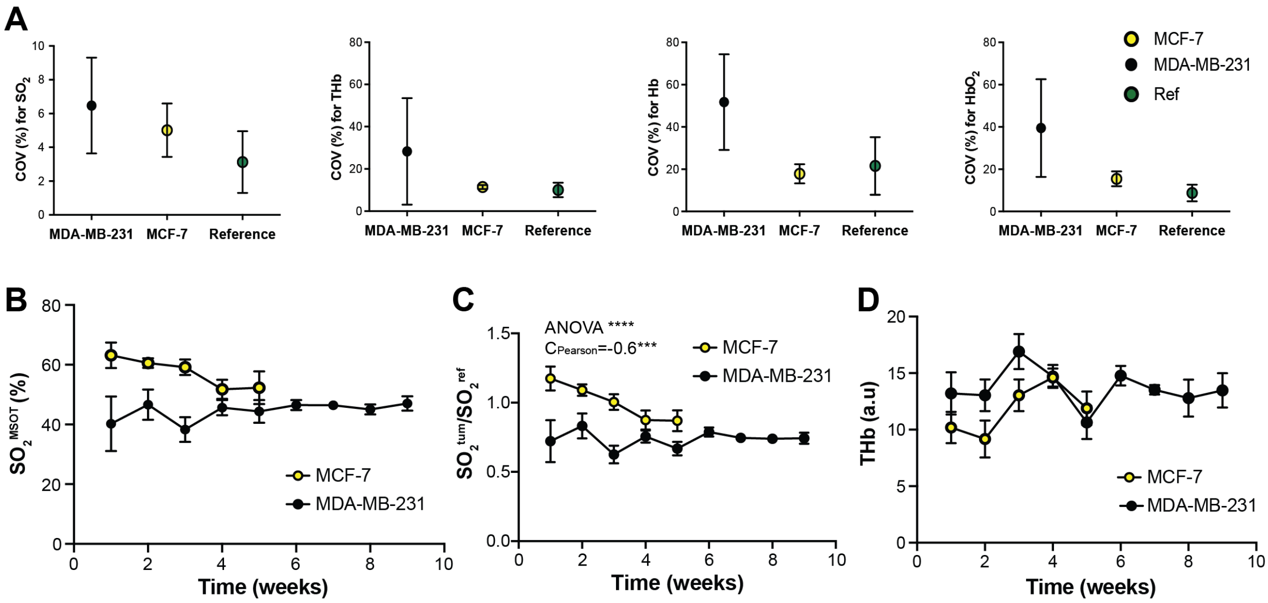
A
COV (%) for SO_2
COV (%) for THb
COV (%) for Hb
COV (%) for HbO_2
MDA-MB-231
MCF-7
Reference
MCF-7
MDA-MB-231
Ref
B
SO_2^{MSOT} (%)
Time (weeks)
MCF-7
MDA-MB-231
C
ANOVA ****
$C_{Pearson}$=-0.6***
SO_2^{tum}/SO_2^{ref}
Time (weeks)
MCF-7
MDA-MB-231
D
THb (a.u)
Time (weeks)
MCF-7
MDA-MB-231

Figure S3: Deoxyhaemoglobin (Hb) and oxyhaemoglobin (HbO_2) signals used to compute total haemoglobin and oxygen saturation in the tumour rim and core. (A, B) Data pooled for all tumours that underwent OT. (C, D) Data from size matched comparison. (E) Rim and core analysis for SO_2^{MSOT} during time, tumours were analysed after they were sufficiently large to enable a 1 mm radius region of interest to be drawn to denote the rim. Statistical significance was assessed by paired t-test within a single tumour type and by unpaired t-test between tumour types. For (A) and (B) and (E) $n^{MCF\text{-}7} = 11$; $n^{231} = 15$, for (C) and (D) $n^{MCF\text{-}7}$ and n^{231} = 4. All panels data expressed as mean±SEM. * $p<0.05$, ** $p<0.01$, *** $p<0.001$, **** $p<0.0001$.

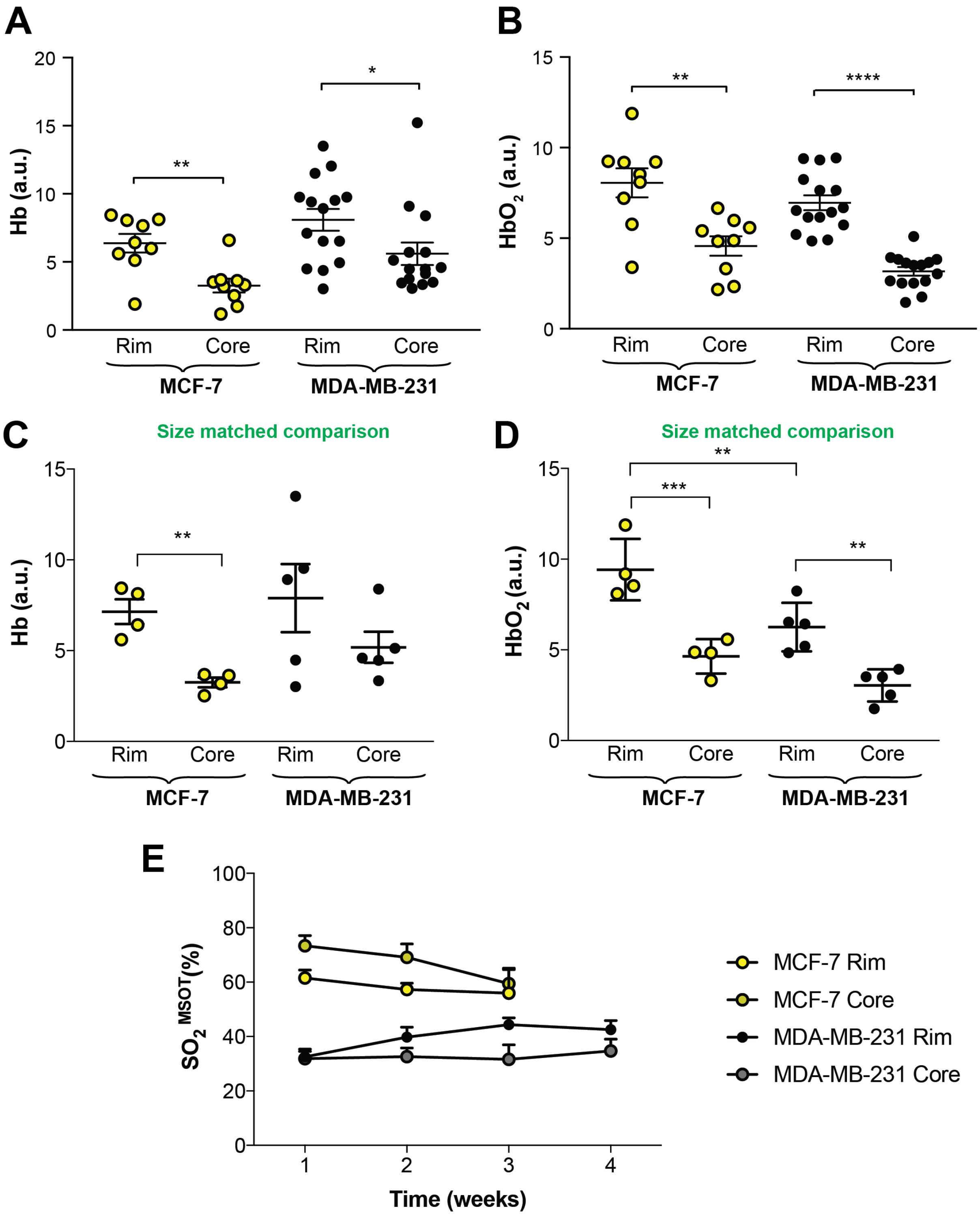
A
Hb (a.u.)
0
5
10
15
20
**
*
Rim
Core
Rim
Core
MCF-7
MDA-MB-231
B
HbO_2 (a.u.)
0
5
10
15
**
****
Rim
Core
Rim
Core
MCF-7
MDA-MB-231
C
Size matched comparison
Hb (a.u.)
0
5
10
15
**
Rim
Core
Rim
Core
MCF-7
MDA-MB-231
D
Size matched comparison
HbO_2 (a.u.)
0
5
10
15
**
***
**
Rim
Core
Rim
Core
MCF-7
MDA-MB-231
E
SO_2^{MSOT}(%)
0
20
40
60
80
100
1
2
3
4
Time (weeks)
MCF-7 Rim
MCF-7 Core
MDA-MB-231 Rim
MDA-MB-231 Core

Figure S4: Inflammatory and oxidative stress markers. (A) Representative image of nitrotyrosilation detection in tumour proteins by western-blot. GADPH was used as a housekeeping protein. (B) Relative quantification normalized against a protein extract of MDA-MB-231 cells treated with $ONOO^-$ at 10 µM. No significant difference was observed between the two models. (C) ELISA quantification of oxidative modification of DNA 8-oxo-dG in tumour tissue. (A) and (B) $n^{MCF\text{-}7}$ = 8; n^{231} = 15. $n^{MCF\text{-}7}$ = 10; n^{231} = 15).

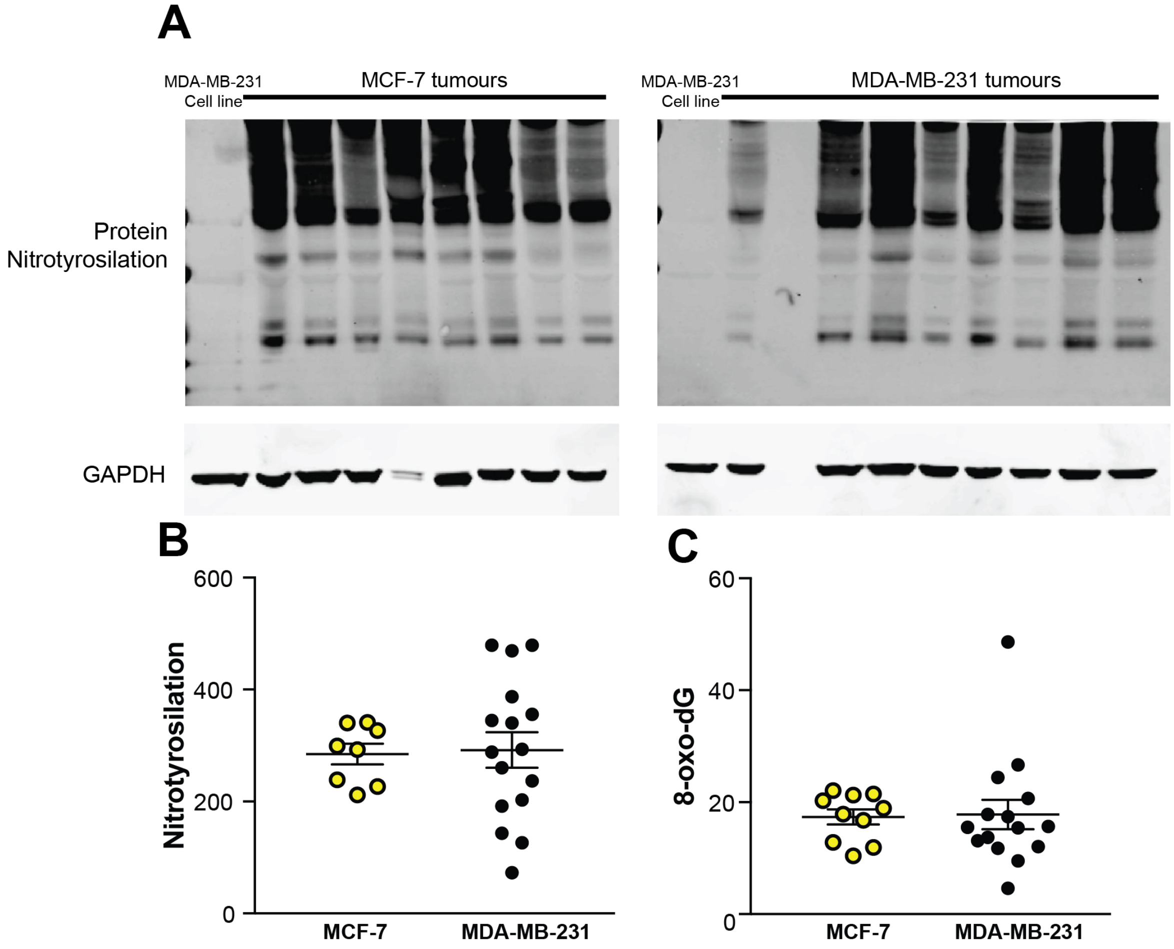

Figure S5: Quantification of PAS and CD31 staining in adjacent sections. (A) Representative micrographs of MCF-7 and MDA-MB-231 tumours (magnification 20x) stained with PAS and immunostained for CD31 in serial sections. Blood vessels were identified in PAS staining (yellow marks) and the corresponding region was located in the CD31 immunostaining. Blood vessels with positive (green) and negative (arrowhead) CD31 staining are marked. (B) Quantification of the CD31+ staining in blood vessels. $n^{MCF\text{-}7}$=6 and n^{231}=6 data expressed as mean±SEM, *$p<0.05$ by unpaired 2-tailed t-test.

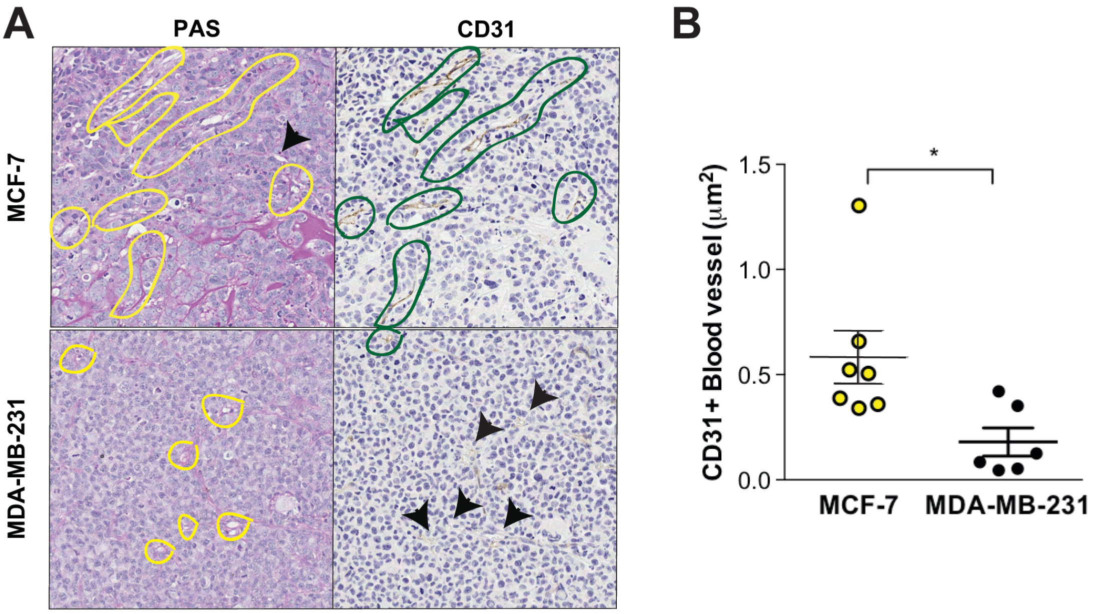

Supplement: Supplementary file 1 — Supplementary Information [file 41416_2018_33_MOESM1_ESM.pdf]
